# Open versus Laparoscopic Surgery: Does the Surgical Technique Influence Pain Outcome? Results from an International Registry

**DOI:** 10.1155/2016/4087325

**Published:** 2016-03-22

**Authors:** Renée Allvin, Narinder Rawal, Eva Johanzon, Ragnar Bäckström

**Affiliations:** ^1^Department of Anaesthesiology and Intensive Care, Örebro University Hospital, 701 85 Örebro, Sweden; ^2^School of Health and Medical Sciences, Örebro University, 701 82 Örebro, Sweden

## Abstract

Postoperative pain management relevant for specific surgical procedures is debated. The importance of evaluating pain with consideration given to type of surgery and the patient's perspective has been emphasized. In this prospective cohort study, we analysed outcome data from 607 patients in the international PAIN OUT registry for assessment and comparison of postoperative pain outcome within the 24 first hours after laparoscopic and open colonic surgery. Patients from the laparoscopic group scored* minimum pain* at a higher level than the open group (*P* = 0.012). Apart from minimum pain, no other significant differences in patient reported outcomes were observed. Maximum pain scores >3 were reported from 77% (laparoscopic) and 68% (open) patients (mean ≥ 5 in both groups). Pain interference with mobilization was reported by 87–93% of patients. Both groups scored high levels of patient satisfaction. In the open group, a higher frequency of patients received a combination of general and regional anaesthesia, which had an impact of the minimum pain score. Our results from registry data indicate that surgical technique does not influence the quality of postoperative pain management during the first postoperative day if adequate analgesia is given.

## 1. Introduction

Providing a high quality pain treatment is a major challenge in health care. Despite the developments in pain management, postoperative pain is still a clinical problem [1–3]. Poorly managed pain reduces quality of life, causes unneeded suffering, and can extend time of discharge from hospital [4]. The importance of evaluating postoperative pain with consideration given to type of surgery and the patient's perspective cannot be overemphasized. Colonic surgery is a common procedure in both female and male patients. Both laparoscopic and open surgeries are performed. Several studies have compared the different surgical techniques with regard to possible benefits of the laparoscopic approach [5, 6].

PAIN OUT started as a European commission-founded project aiming to improve clinical care of patients with postoperative pain [7]. Today patient reported outcomes (PROs) of pain management after a wide range of surgical procedures and audit data from medical records have been collected into an international pain registry [8]. The aim of this prospective clinical study was to analyse patient reported data from the PAIN OUT registry for assessment and comparison of postoperative pain outcome within the 24 first hours after laparoscopic and open colonic surgery.

## 2. Materials and Methodology

### 2.1. Study Design and Samples

In the present cohort study, registry data from patients undergoing laparoscopic and open colonic surgery were included. The registry data had been collected between February 2010 and November 2012 from one to three surgical departments in each of the following countries: France, Germany, Italy, Israel, Romania, Spain, Sweden, Switzerland, and United Kingdom. Inclusion criteria were that patients had to be 16 to 18 years old or older (consenting age varies in the different countries) and could communicate. Patients were excluded if they (I) had been transferred to another ward after surgery; (II) were not present at the ward at the time of data collection; (III) had visitors at the time of data collection; (IV) refused to participate in the study; (V) were sedated or asleep; (VI) had a cognitive dysfunction that precluded complete cooperation. Process data including pre-, intra-, and postoperative variables were collected from the medical records on postoperative day one. On the same day, patients completed an outcome questionnaire, when they were back in the ward for at least 6 hours [9].

The study was planned and implemented based on ethical principles commonly applied in clinical research. All respondents gave their informed consent to enrolment in the study and were guaranteed confidentiality. The study was approved by the institutional review board or ethics committee at all sites. Each patient's oral consent and written consent were obtained before inclusion.

### 2.2. Patient Reported Outcome Measures

Within the PAIN OUT project, the multi-item International Pain Outcomes Questionnaire (IPOQ) was developed. In the IPOQ patients' self-reported outcomes are assessed by an 11-point scale ranging from 0 to 10 (a lower score indicates less problem/difficulty). The questionnaire is presented in detail elsewhere [9]. In this study,* minimum and maximum pain intensity*,* physical and emotional functional interference*, and* perception of care* are presented.

### 2.3. Data Analysis

A descriptive analysis was performed to assess the characteristics of the study sample. The demographic data were described by frequency distribution, mean and range, or mean and standard deviation (SD), respectively, when appropriate. To evaluate differences between the laparoscopic and open surgery groups in demographic variables, chi-square tests were used. Significant differences between the groups in patient outcome data were tested with the nonparametric Mann-Whitney *U* test. A probability of <0.05 was considered statistically significant. The statistical analyses were performed with SPSS 21 (SPSS Inc., Chicago, IL).

## 3. Results

### 3.1. Demographic and Characteristics of the Sample

A total of 619 patients who underwent colonic surgery were identified from the PAIN OUT registry. Twelve patients (2%) were excluded because the procedure was changed from laparoscopic to open surgery. From the remaining 607 patients, 450 (74%) had undergone open surgery and 157 (26%) had undergone laparoscopic surgery. The most frequent localization of surgery was right and left hemicolectomy. Patient demographic data and intraoperative characteristics are presented in Table 1.

### 3.2. Patient Reported Outcome

**Figure 1:**
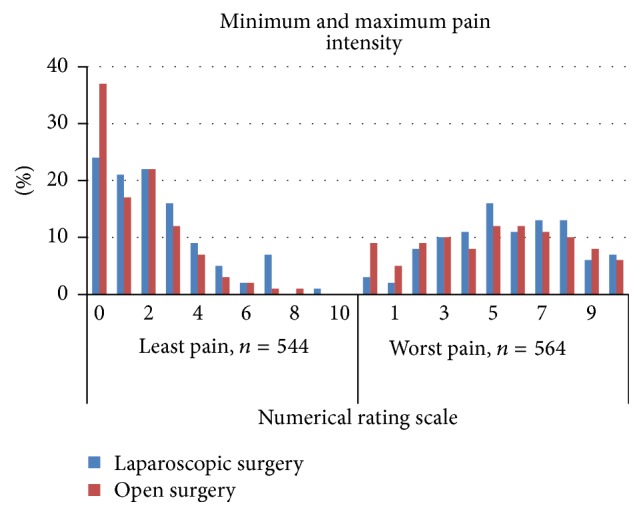
Distribution of the scores for* minimum* and* maximum pain* within 24 hours after laparoscopic and open colonic surgery (NRS 0 = no pain to 10 = worst pain possible).

**Figure 2 fig2:**
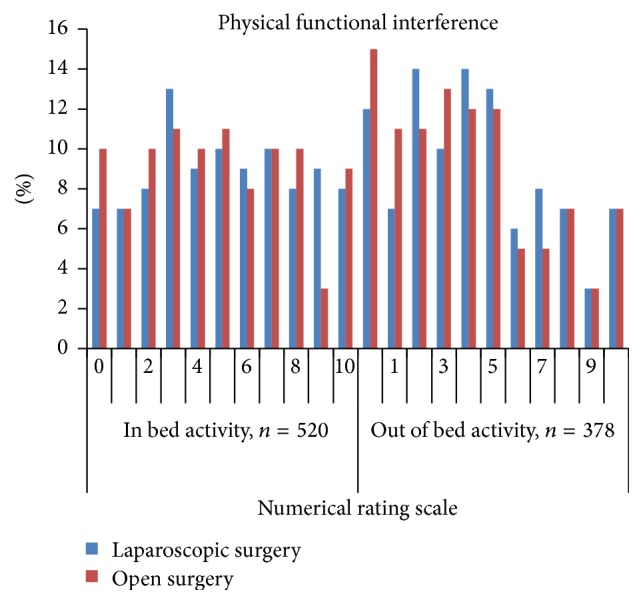
Distribution of the scores for pain* interfering with activities* in bed and out of bed within 24 hours after laparoscopic and open colonic surgery (NRS 0 = did not interfere to 10 = completely interfered).

**Figure 3 fig3:**
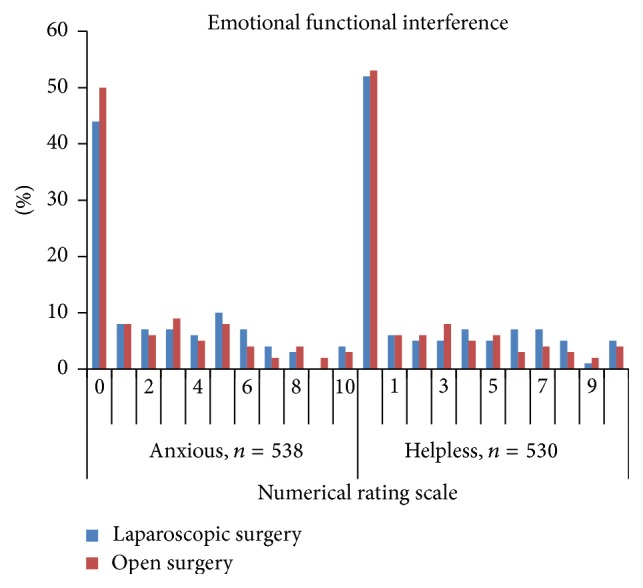
Distribution of the scores for pain interfering with* feeling anxious* and* helpless* within 24 hours after laparoscopic and open colonic surgery (NRS 0 = not at all to 10 = extremely).

**Figure 4:**
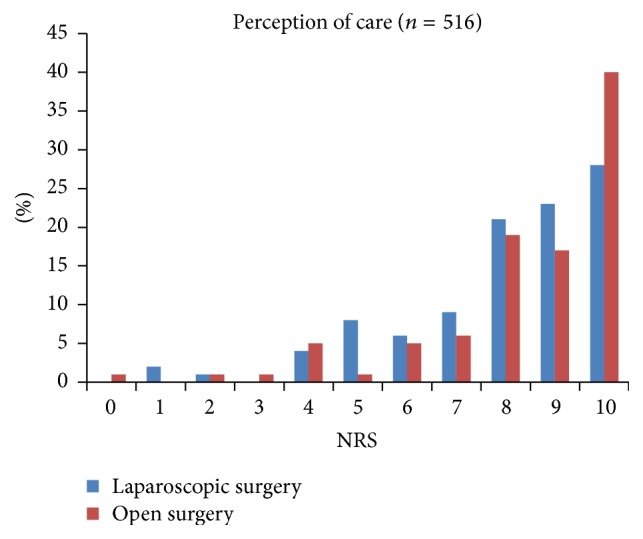
Distribution of the scores for* patient satisfaction* with the result of pain management within 24 hours after laparoscopic and open colonic surgery (NRS 0 = extremely dissatisfied to 10 = extremely satisfied).

Patient's self-assessments of pain management outcomes on the first postoperative day are presented in Figures 1 [Fig fig2] [Fig fig3]–4.

### 3.3. Comparisons

A significant difference was seen between the groups in the distribution of age and anaesthesia technique. In the laparoscopic surgery group, there was a higher frequency of patients in the age group ≤65 years (*P* = 0.018). In the open surgery group, there was a higher frequency of patients receiving a combination of general and regional anaesthesia (*P* = 0.01) (Table 1). No significant differences were seen between the groups regarding the frequency of patients that had been mobilized out of bed during the first 24 hours. The frequency of patients with maximal pain scores >3 was 70% for all patients, 77% for laparoscopic surgery, and 68% for open surgery (no significant difference between the groups).

Only one item in the IPO questionnaire showed a significant difference between laparoscopic and open surgery. The patients from the laparoscopic group scored* minimum pain* at a higher level than the open surgery group (*P* = 0.012) (Table 2). Repeated statistical analyses showed that the difference in* minimum pain* between the surgery groups remained significant when controlling for age, but anaesthesia technique had an impact. There was a difference in pain score between the surgery groups if the patients received a combination of general and regional anaesthesia (*P* = 0.009), but no difference if general anaesthesia was used alone (*P* = 0.42).

## 4. Discussion

The findings of this cohort study are based on data from an international pain registry. In the comparison of patient outcomes within 24 hours after laparoscopic and open colonic surgery, the laparoscopic group scored a significantly higher level of minimum pain. Although statistically significant, these values do not have much clinical importance. Apart from minimum pain, no other significant differences in patient reported outcomes were observed. Postoperative pain management relevant to specific surgical procedures is debated since the efficacy of different analgesic approaches varies between different surgical procedures [10]. A higher frequency of patients from the open surgery group received both general anaesthesia and regional anaesthesia, which had an impact on reported minimum pain score. It is generally believed that laparoscopic technique is less painful than the open one but clearly it will depend on how pain is managed. Our results from over 600 patients from one to three centres in nine countries suggest that if pain is managed adequately, the choice of surgical technique is not so relevant.

Generally, postoperative pain constitutes an important issue for patients undergoing surgery [11]. According to surgical and anaesthesiology personnel, colonic surgery patients often express concerns about pain and the expected level of pain intensity [12]. In this study, a majority of patients in both groups reported maximum pain score at NRS >3 (mean ≥ 5 in both groups). These results correspond with those from a study by Gerbershagen et al. [13]. Furthermore, 87–93% patients in our study reported pain interference with activities both in bed and out of bed. Nonetheless, high satisfaction with the pain management and low levels of emotional interference were reported. It has been argued that the goal of any pain treatment should be its ability to improve perioperative outcome and mobilization rather than achieving a specific pain score [14].

During the last decade, the use of PROs has been introduced to quality registries [15]. Compared to clinical outcomes, which remain the primary endpoints for most clinical trials, PROs often carry more meaning for patients affected by an intervention [16]. Registry data within a population makes it possible to have a strict focus on outcome from a patient perspective [17]. Registry data can be actively used for increasing the person centeredness of postoperative pain management. PAIN OUT constitutes a unique information source and benchmarking instrument. To our knowledge, it is the only international registry with standardized assessments of pain management after routine surgical interventions. Compared to randomized controlled studies, with strict inclusion and exclusion criteria, registry studies reflect the “real world.” A further strength of this study is the comparatively large population size.

A limitation is that we did not have access to data concerning the patients who were excluded. Given the large number of exclusion criteria, for example, patients who were not present at the ward, were in too much pain or too ill, and did not want to participate are not represented in the registry, thereby introducing a potential source of bias. Furthermore, the assessment of postoperative pain and pain management was performed only within the first 24 hours after the surgical intervention. Patients experience pain of varying levels during a number of days after colonic surgery [18]. However, we do not know if pain intensity differs between the groups after 24 hours.

The overall planning of postoperative care and nursing interventions should be performed out of a procedure specific perspective. Comparisons of laparoscopic and open colonic surgery have been performed in the nursing literature. Nurses working in a specialist colorectal unit perceived improved outcomes in terms of lower pain intensity among patients who had undergone laparoscopic surgery. They also perceived that it took less effort to care for the laparoscopic patients [19]. In order to improve the quality of postoperative care, it would be of interest to compare these findings with patient outcome data. For example, it is well known that nurses often misjudge the pain intensity that patients are experiencing [20].

More frequent administration of analgesics for mild pain after laparoscopic surgery was reported in a retrospective study, suggesting that laparoscopic surgery is less painful [21]. However, retrospective reviews of medical records, with documentation of delivered analgesics, do not necessarily reflect patients' self-perception of pain intensity. If laparoscopic procedures generally are rated as less painful, patients undergoing this type of surgery run the risk of receiving inadequate pain relief. This has been pointed out in a comprehensive cohort study demonstrating unexpectedly high levels of postoperative pain after laparoscopic surgeries, with absence or low levels of analgesic use [12]. Nursing responsibilities in clinical practice include providing individualized surgical care helping patients to return to everyday life [22]. An essential part of the patient's postoperative care is pain management relevant to the specific surgical procedure in question [13]. PROs from registry data reflect clinical reality and could form a base for the development of caring plans and evaluation of nursing and medical interventions.

## 5. Conclusion

According to registry, clinical data surgical technique does not influence the quality of postoperative pain management during the first postoperative day if adequate analgesia for the procedure in question is given.

## Figures and Tables

**Table 1:** Demographic data (frequencies, mean, range, SD, and chi-square test) of the participants and of intraoperative characteristics.

	Open surgery	Laparoscopic surgery	Chi-square test *P* value
Sex, *n* (%)			0.712
Female	198 (44)	71 (45)	
Male	251 (55)	84 (54)	
Unknown	1 (1)	2 (1)	

Age (years), mean (SD)	63 (15)	60 (14)	
Age, group			0.018^*∗*^
18–65, *n* (%)	223 (49)	95 (60)	
>65, *n* (%)	224 (50)	61 (39)	
Unknown	3 (1)	1 (1)	

Type of surgery^1^, *n* (%)			
Cecectomy	43 (7)	4 (1)	
Right hemicolectomy	188 (31)	42 (7)	
Resection transverse colon	11 (2)	18 (3)	
Left hemicolectomy	51 (8)	10 (2)	
Sigmoidectomy	101 (17)	70 (12)	
Unspecified excision LI^2^	56 (9)	13 (2)	

Duration of surgery			
Mean (range), min	173 (30–695)	182 (40–600)	0.573

Anaesthesia technique			<0.01^*∗*^
GA, *n* (%)	169 (37)	87 (58)	
GA + RA, *n* (%)	275 (61)	63 (42)	
Unknown	6 (2)		

^1^ICD-9 classification.

^2^LI = large intestine.

^*∗*^ *P* ≤ 0.05.

**Table 2:** Frequencies, mean, SD, and significant difference for type of surgery.

Item	Open surgery			Laparoscopic surgery			Mann-Whitney *U* test
	*N*	M	SD	*N*	M	SD	*P* value
Worst pain^1^	404	5.08	2.91	142	5.56	2.55	0.131
Least pain^1^	403	1.61	1.72	141	1.97	1.74	0.012^*∗*^
Pain interfering with activities in bed^2^	385	4.75	3.05	135	5.04	3.03	0.345
Pain interfering with activities out of bed^2^	261^§^	3.93	3.00	117^§^	4.27	2.94	0.261
Being anxious^3^	399	2.23	2.91	139	2.55	2.97	0.207
Being helpless^3^	396	2.13	2.95	134	2.40	3.14	0.475
Being satisfied with the result of pain treatment^4^	380	8.29	2.03	136	8.04	2.00	0.076

^1^0 = no pain to 10 = worst pain possible; ^2^0 = did not interfere to 10 = completely interfered; ^3^0 = not at all to 10 = extremely; ^4^0 = extremely dissatisfied to 10 = extremely satisfied.

^§^Lower total *n* because this item was scored only by the patients that had been out of bed.

^*∗*^ *P* ≤ 0.05.
